# Cell-to-Cell and Patient-to-Patient Variability in Antimicrobial Resistance

**DOI:** 10.3390/microorganisms13122766

**Published:** 2025-12-04

**Authors:** Xiaoyun Huang, Junjie Huang, Claire Chenwen Zhong, Martin C. S. Wong

**Affiliations:** 1 JC School of Public Health and Primary Care, Faculty of Medicine, The Chinese University of Hong Kong, Hong Kong SAR, China; junjiehuang@cuhk.edu.hk (J.H.); chenwenzhong@cuhk.edu.hk (C.C.Z.); 2 Center for Health Education and Health Promotion, Faculty of Medicine, The Chinese University of Hong Kong, Hong Kong SAR, China; 3 School of Public Health, Peking University, Beijing 100191, China; 4 School of Population Medicine and Public Health, Chinese Academy of Medical Sciences and Peking Union Medical College, Beijing 100730, China; 5 School of Public Health, Fudan University, Shanghai 200032, China

**Keywords:** variability, antimicrobial resistance, single cell, persister, bet hedging

## Abstract

Antimicrobial resistance (AMR) remains a global health crisis, yet treatment outcomes cannot be explained by resistance genes alone. Increasing evidence highlights the importance of variability at two levels: within bacterial populations and across patients. At the microbial level, cell-to-cell variability including genetic mutations, stochastic gene expression, persister cell formation, heteroresistance, and spatial heterogeneity within biofilms creates phenotypic diversity that allows subsets of bacteria to survive antimicrobial stress. At the host level, patient-to-patient variability including differences in genetic background, immune competence, comorbidities, gut microbiome composition, and pharmacokinetics shapes both susceptibility to resistant infections and the likelihood of treatment success. Together, these dimensions explain why infections with the same pathogen can lead to divergent clinical outcomes. Understanding and integrating both microbial and host variability offers a path toward more precise diagnostics, personalized therapy, and novel strategies to counter AMR.

## 1. Introduction

Antimicrobial resistance (AMR) in bacteria is a growing threat to global health, yet treatment failure is not solely due to acquired resistance genes [1]. Even infections with bacteria lacking traditional resistance mutations can fail to resolve under antibiotic therapy [2]. Two often underappreciated dimensions contribute to this phenomenon: (1) cell-to-cell variability among bacteria, meaning that genetically identical bacteria can exhibit heterogeneous phenotypes that affect drug susceptibility; (2) patient-to-patient variability, where host factors lead to different infection outcomes. In this review, we examine recent insights into how microbial heterogeneity and host variability intersect to influence antimicrobial treatment outcomes. Both laboratory studies and clinical evidence are highlighted, including key mechanisms, illustrative case studies, and emerging strategies connecting bacterial phenotypic heterogeneity and host differences to AMR-related treatment outcomes.

## 2. Bacterial Cell-to-Cell Variability and Antimicrobial Resistance

**Figure 1:**
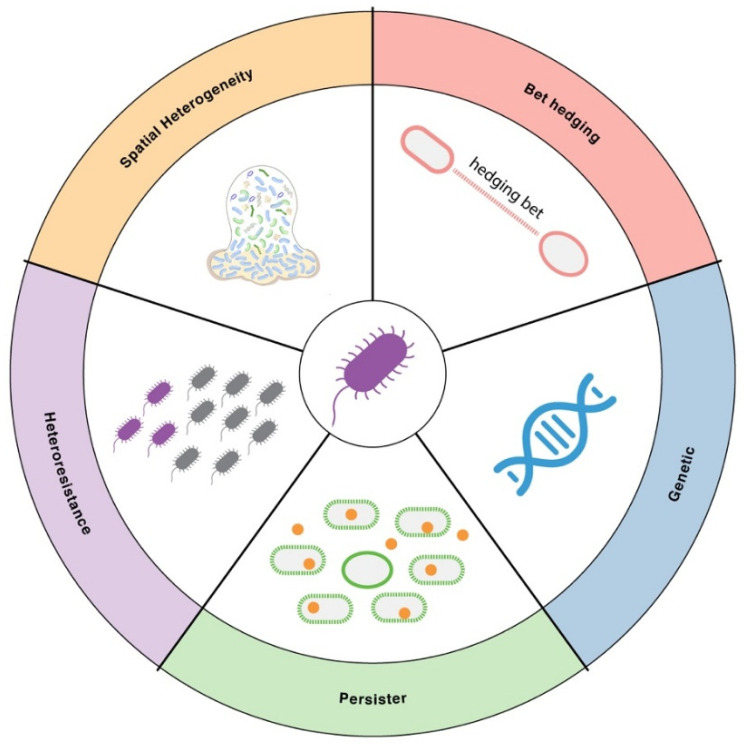
Major sources of cell-to-cell variability in AMR. Illustration of key sources of bacterial cell-to-cell variability in AMR. These include genetic variation, bet-hedging through stochastic gene expression, formation of dormant persister cells, heteroresistance arising from resistant subpopulations within an isogenic culture, and spatial heterogeneity in biofilms or microenvironments.

Even within a genetically identical bacterial population, individual cells can respond very differently to antibiotics. This heterogeneity arises from multiple sources, including spontaneous genetic changes, stochastic gene expression (“bet-hedging”), the formation of dormant persister cells, the presence of heteroresistant subpopulations, and spatial variation within biofilms (Figure 1). These forms of variability allow some cells to survive antimicrobial stress while others are killed, creating reservoirs for relapse and complicating treatment outcomes. Below, we explore key bacterial variability mechanisms that shape antimicrobial resistance.

### 2.1. Phenotypic Heterogeneity and “Bet-Hedging” in Bacterial Populations

Bet-hedging is an evolutionary strategy by which genetically identical organisms generate phenotypic heterogeneity to ensure that a subset of the population survives unpredictable or fluctuating environmental stresses, such as antibiotic exposure [3,4,5]. The mechanisms underlying this process can be stochastic or regulated. Isogenic bacterial populations can display remarkable phenotypic heterogeneity owing to stochastic gene expression (“noise”) and regulatory network architecture [6]. Random fluctuations in mRNA/protein levels can push individual cells into distinct states—a bet-hedging strategy whereby a fraction of cells can survive environmental stress (like antibiotics) while the rest perish [7]. This non-genetic diversity means that when an antibiotic is applied, some cells may be in a protected physiological state, whereas their genetically identical siblings are not. Over the past decade, studies combining single-cell imaging and modeling have shown that such phenotypic variation significantly impacts antibiotic responses: despite uniform drug exposure, some cells die while others continue to grow. These chance survival events complicate therapy and can alter the evolutionary trajectory of resistance in a bacterial population. Importantly, phenotypic heterogeneity is not just “noise”—there is evidence bacteria actively exploit it. For example, positive feedback circuits in regulatory networks can create bistable states, yielding distinct subpopulations that persist over generations [8]. Recent work in *E. coli* demonstrated that cell survival under β-lactam antibiotic stress can be lineage-correlated [9]. If a parent cell survives, its progeny is more likely to survive—indicating transiently heritable phenotypic resistance factors. In this study, “robust lineages” emerged within an otherwise susceptible population, driven by intergenerational memory of a stress-tolerant physiological state. These findings underscore that phenotypic heterogeneity, far from being merely random, can propagate antibiotic survival traits through a bacterial lineage. Emerging omic technologies with single cell resolution are leveraged to investigate the interplay of gene expression variation and phenotype variability [10,11].

The phenotypic heterogeneity is also tightly regulated. It was shown that *Mycobacterium* actively generates cell-to-cell heterogeneity through the divisome protein LamA, which drives asymmetric polar growth and produces subpopulations with differential antibiotic susceptibility, thereby enhancing survival under drug pressure [12]. Many antibiotics rely on active metabolic processes to exert their killing effect, bacteria in low-metabolic or metabolically altered states can survive drug exposure, making metabolic state a key driver of phenotypic antimicrobial resistance [13,14,15]. Several studies highlight how bacterially derived gaseous messengers such as NO and H_2_S, along with host-driven redox perturbations, create physiological states that enhance antibiotic tolerance, underscoring metabolic and redox regulation as central contributors to antimicrobial resistance [16,17,18].

### 2.2. Genetic Variability

Genetic variability is a major source of cell-to-cell variability in AMR [19,20,21]. It was demonstrated that machine learning models built on whole-genome sequencing data can predict antibiotic resistance more accurately than relying on single resistance SNPs alone [22]. By training support vector machine classifiers on genomic features, the researchers achieved perfect specificity and markedly improved sensitivity for predicting resistance to clarithromycin and amoxicillin compared to traditional SNP-based markers. Antibiotic resistance is not always reducible to a single mutation. Instead, it often reflects combinatorial effects of multiple loci, gene regulation, and epistatic interactions, which contribute to phenotypic variability at the single-cell level. Thus, predictive modeling that integrates broader genomic information aligns with the broader recognition that variability in bacterial populations—both genetic and phenotypic—complicates treatment outcomes. The AMR related genomic data has been archived in public database to increase access [23]. By moving beyond single markers, such approaches better capture the complexity of resistance expression, bridging the gap between genetic heterogeneity and cell-to-cell phenotypic variability in antimicrobial response.

It was found that consistent patterns of global epistasis between plasmids and bacterial genomes can make plasmid–host associations partially predictable, suggesting that understanding these genetic interaction rules may help forecast which antimicrobial-resistance plasmids are most likely to spread successfully in the future [24]. The AMR genomic features differ in different climates and geographic environments. A global metagenomic atlas of urban mass-transit systems across 60 cities was generated, revealing thousands of previously uncharacterized microbes and extensive geographic variation in antimicrobial resistance genes [25].

By targeting bacterial evolvability factors such as the DNA translocase Mfd [26,27,28], which drives mutagenesis and accelerates resistance emergence, inhibiting the mutation rate itself offers a promising anti-evolution strategy to slow or prevent antimicrobial resistance. Furthermore, several studies demonstrate that targeting bacterial mutagenesis pathways—including the SOS response [29,30,31]—offers a promising anti-evolution strategy to slow or prevent the emergence of antimicrobial resistance during therapy.

### 2.3. Persister Cells and Antibiotic Persistence

**Figure 2:**
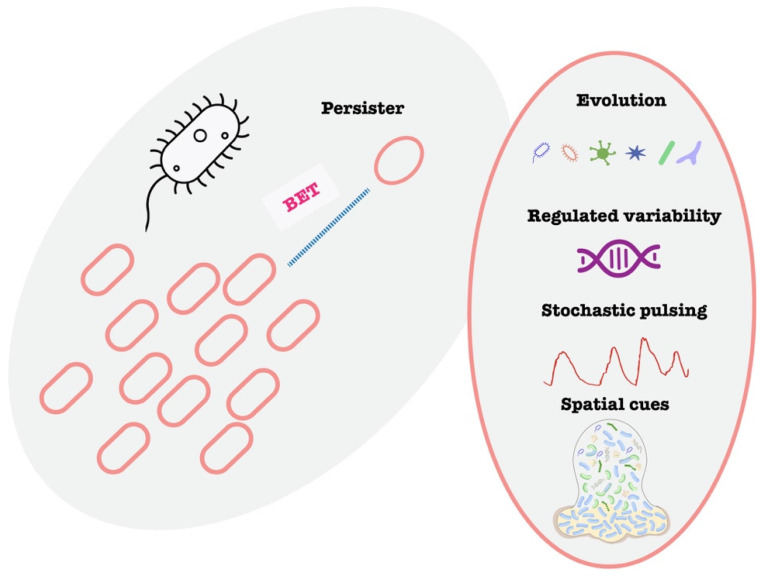
Schematic illustration of bet-hedging and persister. Illustration of bet-hedging and the variable mechanisms to generate persistence.

Persisters are a classic example of cell-to-cell variability impacting AMR (Figure 2). Persisters are a subpopulation of phenotypically variant, non-replicating or slow-growing bacterial cells that transiently tolerate lethal concentrations of antibiotics without acquiring heritable resistance and can resume growth once the antibiotic pressure is removed [32,33,34,35]. The phenomenon was discovered by Gladys Hobby in 1942 and the term “persister” was proposed by Joseph Bigger in 1944 [36]. Persister cells are a small subset of a bacterial population that enter a dormant, non-dividing state, allowing them to tolerate antibiotics that require active growth to kill [37]. They do not have genetic resistance, but they survive high antibiotic doses that kill off the majority of their peers [38]. Once the antibiotic is removed, persisters can “wake up” and repopulate, leading to recurrence of infection. Persisters have been observed in virtually all major bacterial species and are induced by various stress responses. Persisters are clinically significant because they underlie many chronic and hard-to-treat infections. A famous example is tuberculosis: the long duration of TB therapy (6+ months) is largely needed to eradicate persister bacilli that survive initial bactericidal treatment, otherwise the disease relapses [39]. Similarly, *Staphylococcus aureus* persisters contribute to recurrent infections like endocarditis and osteomyelitis despite adequate antibiotic courses [40]. Laboratory and animal model studies confirm persisters’ role in treatment failure: infections seeded with persister cells show delayed clearance and frequent relapse, whereas normal cells are cleared by the same treatment. Notably, persisters also exhibit evasion of host immunity—their dormant state makes them less detectable and susceptible to phagocytic killing and other innate defenses. In one study, *Pseudomonas aeruginosa* persister infections in model hosts led to diminished early immune activation compared to regular cells [41]. This “stealth” characteristic contributes to chronic, smoldering infections. Taken together, persister cells represent a phenotypic hedge that bacteria use to survive transient stress, linking single-cell variability to clinical persistence of infection.

Recurrent urinary tract infections (UTIs) caused by drug-susceptible *Escherichia coli* exemplify the clinical impact of bacterial persistence [42]. Despite appropriate antibiotic therapy, patients frequently experience relapse within weeks, driven by subpopulations of dormant persister cells that evade treatment by residing intracellularly within bladder epithelial cells or remaining quiescent in the urinary tract. Upon cessation of antibiotic exposure, these persisters can reactivate and reinitiate infection [43]. Similar dynamics are observed in *Staphylococcus aureus* infections of bone and soft tissue, where treatment failure is linked to a residual pool of non-replicating cells [44]. These clinical scenarios underscore the need for therapeutic strategies targeting both actively dividing and phenotypically tolerant bacterial subpopulations.

### 2.4. Heteroresistance and Phenotypic Resistance

While persistence and tolerance involve transient antibiotic tolerance, heteroresistance refers to a mixed susceptibility phenotype: within a clonal bacterial population, a subpopulation exhibits a much higher MIC to an antibiotic than the rest [45]. In heteroresistance, a fraction of cells behave as if they are fully resistant, even though the majority are susceptible. This phenomenon can lead to treatment failure or diagnostic confusion: in a laboratory susceptibility test, the bulk of the culture might appear susceptible, but a minor resistant subpopulation can expand under therapy. In *Enterobacter cloacae*, cationic antimicrobial peptide (CAMP) heteroresistance is driven by PhoPQ-dependent, PmrAB-independent lipid A modification with l-Ara4N, revealing a distinct regulatory mechanism contributing to colistin resistance in this emerging nosocomial pathogen [46]. Standard MIC testing might classify such an isolate as “susceptible”, potentially leading to inappropriate therapy. Indeed, heteroresistance is often missed by routine diagnostics and requires specialized population analysis to detect. Mechanistically, heteroresistance can arise from unstable genetic events that increase the copy number of a resistance gene in a subset of cells [47], which can revert once drug pressure is removed or phase variation [48].

Heteroresistant infections behave unpredictably. The population may respond to antibiotics initially, then rebound as the resistant subclone takes over. Growing awareness of this is leading researchers to argue that even small resistant subpopulations must be considered in treatment design. In fact, mounting clinical evidence indicates that both persistence and heteroresistance can cause antibiotic treatment failure. For example, heteroresistance in *Klebsiella pneumoniae* and *E. cloacae* has been linked to patient treatment failures and is reproducibly observed in animal infection models [49]. Consequently, calls have been made to update diagnostic protocols to detect these minority phenotypes, rather than relying solely on bulk population MIC.

### 2.5. Biofilms and Spatial Heterogeneity

An important contributor to cell-to-cell variability in drug susceptibility is the formation of biofilms—structured communities of bacteria encased in a protective matrix [50]. Biofilms create steep chemical gradients within their structure, yielding heterogeneous microenvironments. Cells deep in a biofilm are often slow-growing or starved, which confers high antibiotic tolerance since many antibiotics target active metabolic processes [51]. The biofilm matrix impedes antibiotic penetration, allowing embedded bacteria to survive concentrations lethal to planktonic cells. In cystic fibrosis, *Pseudomonas aeruginosa* forms biofilms in airway mucus where antibiotics lose efficacy despite appearing potent in vitro [52]. Standard susceptibility tests using planktonic cultures may therefore overestimate drug effectiveness. Biofilms also foster phenotypic variants such as persisters and small-colony variants, contributing to treatment failure and chronic infection. Biofilms also promote the emergence of persisters and small-colony variants within them [53]. Therefore, bacterial lifestyle is a key factor in phenotypic heterogeneity. Multiple clinical studies have documented cases where infections involving biofilms required extended or combination antibiotic regimens, or ultimately failed treatment, despite the absence of detectable resistance genes [54]. These therapeutic failures are often due to phenotypic tolerance conferred by the biofilm environment, including reduced growth rates, altered metabolism, and restricted antibiotic penetration [55,56,57]. In response, current research is increasingly developing advanced infection models and diagnostic tools that incorporate biofilm-specific conditions and tolerance mechanisms, aiming to improve the predictive value of antimicrobial susceptibility testing for real-world clinical outcomes.

Using bacterial single-cell RNA-seq, *S. aureus* biofilm cells were profiled, which reveal that they segregate into multiple transcriptional clusters, each expressing different sets of genes despite being the same strain [58]. Advanced imaging technology was introduced to capture all cells in a biofilm at single-cell resolution over time, even in anaerobic interiors [59]. Another area of innovation is microfluidic and lab-on-chip devices that allow precise control of the biofilm’s environment [60]. Microfluidic biofilm reactors can grow biofilms under defined flow, nutrient, and antibiotic gradients while observing them in real-time by microscopy. These technological advances are enabling researchers to move from descriptive observations of heterogeneity to mechanistic understanding.

## 3. Patient-to-Patient Variability in AMR Outcomes

**Figure 3:**
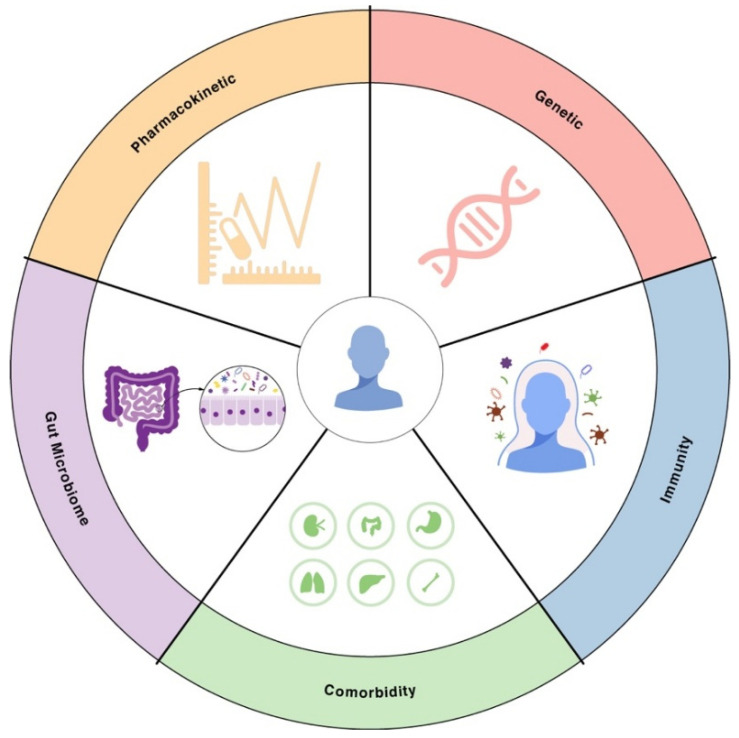
Sources of patient-to-patient variability in AMR. Illustration of key sources of patient-to-patient variability in AMR. These include human genetic background, immune competence, comorbidities, differences in gut microbiome composition that affect colonization resistance, and interindividual pharmacokinetics determining antibiotic exposure.

While bacterial factors are critical, the host context can make the difference between treatment success and failure, especially in infections caused by drug-resistant microbes. Patients vary widely in their immune capabilities, microbiomes, underlying diseases, and how their bodies process drugs (Figure 3). These factors can modulate both the likelihood of encountering an AMR pathogen and the outcome of an AMR infection. In following sections, we explore key host variability factors, including immunity, comorbidity, gut microbiome, pharmacokinetic factors.

### 3.1. Immune System Differences and Comorbidities

A patient’s immune competence is a major determinant of infection outcome, particularly when facing antimicrobial-resistant bacteria. A robust immune system can compensate partially for antibiotic shortcomings—for example, clearing residual bacteria that survive antibiotic exposure. Conversely, immunocompromised patients often experience worse outcomes with AMR infections. Without immune “backup”, even a small number of resistant or tolerant bacteria can proliferate unchecked. Indeed, severely immunocompromised hosts provide a favorable environment for resistance to emerge, since there is minimal immune-mediated bacterial clearance or competition. Clinical studies consistently show higher morbidity and mortality from drug-resistant infections in patients with weakened immunity. For example, hospitalized patients with febrile neutropenia who contract a Gram-negative infection fare significantly worse if the organism is multi-drug resistant versus susceptible. It was shown that the majority of multidrug-resistant infections now originate in the community, underscoring the importance of host factors—such as immune competence—in determining infection risk and clinical outcomes across diverse settings [61]. This stark difference reflects both delays in effective therapy and the host’s inability to control the infection without help. Another host factor is age [62]—the very young and very old have weaker or less coordinated immune responses. Older adults (particularly men) were projected to bear a disproportionate burden of antimicrobial-resistant bloodstream infections in the coming decades, highlighting the critical need for age and sex specific strategies in AMR control.

Taking macrophage as an example, macrophages play a central role in antimicrobial resistance by inducing a drug-tolerant state in *Mycobacterium tuberculosis*, in part by triggering efflux pump–mediated rifampicin tolerance within the intracellular environment [63]. It was shown that intracellular heterogeneity within macrophages—where antibiotics distribute unevenly across lipid droplets and diverse mycobacterial compartments—creates variable drug exposure that contributes to antimicrobial resistance and treatment failure [64]. Macrophage functional heterogeneity creates diverse intracellular microenvironments that impose uneven stress on genetically identical bacteria, promoting the formation of antibiotic persisters and thereby contributing to antimicrobial resistance even in the absence of genetic resistance [65]. Besides macrophage, other immune cell type also play an important role in antimicrobial response and resistance, including monocyte [66], neutrophil [67], dendritic cell [68], T cell [69,70], NK cell [71] and plasma cells [72].

Host genetic background plays a pivotal role in determining susceptibility to infections and the outcome of antimicrobial therapy. Polymorphisms in genes involved in innate and adaptive immunity—such as those encoding Toll-like receptors [73,74,75], cytokines [76,77], and pattern recognition receptors [78]—can alter immune activation thresholds, inflammatory responses, and pathogen recognition, ultimately influencing how effectively an infection is controlled. For example, certain polymorphisms in the *IL-10* promoter are associated with uncontrolled infection and sudden infant death [79]. In the context of antimicrobial resistance, this interindividual variability means that two patients infected with the same pathogen and treated with the same drug may experience vastly different outcomes. Understanding host genetic factors is therefore essential for advancing personalized infectious disease management and for developing therapies that account not only for microbial but also for host-driven heterogeneity.

Underlying comorbidities can also predispose to resistant infections and worse outcomes. One prominent example is diabetes mellitus. Chronic hyperglycemia in diabetics can impair neutrophil and macrophage function and create tissue environments that favor bacterial growth. A systematic review encompassing 61 studies and 449,247 subjects found that diabetic patients had more than double the odds of having a resistant UTI or pneumonia compared to non-diabetics [80]. Clinical observations support that diabetic foot infections, for example, are frequently polymicrobial and often involve Methicillin-resistant *Staphylococcus aureus* (MRSA) or other MDR organisms [81]; poor peripheral circulation and high glucose levels can diminish antibiotic penetration and leukocyte function, leading to suboptimal response. Aside from diabetes, other chronic conditions—such as chronic kidney disease, chronic lung diseases like cystic fibrosis or COPD, and liver disease—can modulate outcomes. For instance, patients with liver cirrhosis in ICUs have very high rates of colonization and infection with MDR bacteria, and this is linked to increased mortality and reduced transplant-free survival. In one cohort, one-third of ICU patients with cirrhosis were colonized by at least one MDR pathogen on admission, and those colonized had a significantly higher risk of developing actual infection and a worse prognosis [82].

Individuals vary widely in their baseline ability to fight infections. Hosts with strong, intact immune systems may successfully clear infections that are only partially suppressed by antibiotics, whereas immunocompromised or medically complex patients often cannot, leading to prolonged infections or death when resistant pathogens are involved. Host-specific factors such as age-related immune decline, immunosuppression, and underlying comorbidities significantly influence the clinical trajectory and treatment outcomes of infections caused by antimicrobial-resistant pathogens [83,84,85]. These variables can impair pathogen clearance and reduce the efficacy of antimicrobial therapy, underscoring that effective AMR management must account for both microbial and host determinants. Consequently, there is growing interest in adjunctive therapeutic strategies—such as immunomodulators, cytokine therapies, or microbiome-targeted interventions—that enhance host defense mechanisms and improve outcomes in patients with resistant infections [86].

### 3.2. Gut Microbiome Variability and Colonization Resistance

**Figure 4:**
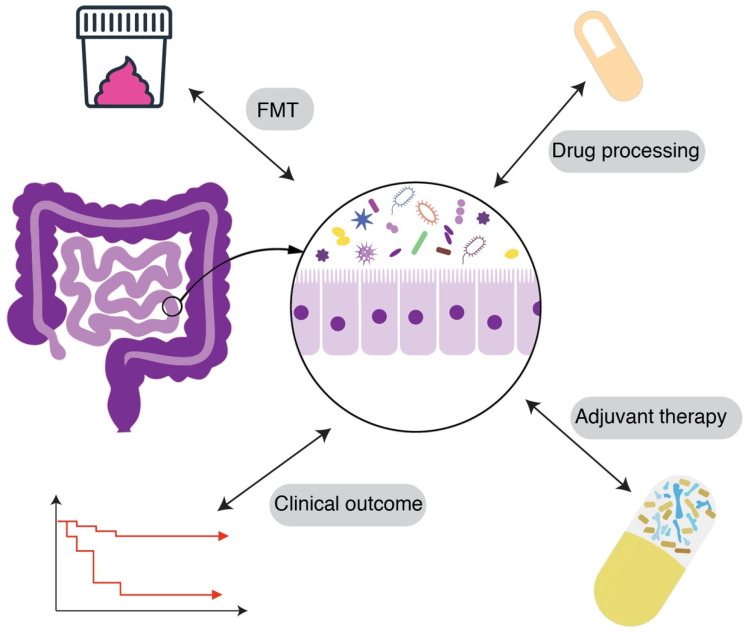
Gut microbiome variability and colonization resistance. Illustration of the complex gut microbiome variability and potential intervention strategies. FMT: Fecal Microbiota Transplantation.

The human microbiome—particularly the gut flora—is another critical piece of the patient-to-patient variability puzzle (Figure 4). The composition of microbiota can influence both the risk of acquiring resistant organisms and the efficacy of antibiotic treatments [87]. A diverse, healthy gut microbiome typically exhibits colonization resistance—the ability to prevent pathogenic bacteria from establishing a colony [88]. Conversely, microbiome disruption can predispose patients to colonization by opportunistic MDR organisms like *Clostridioides difficile*, vancomycin-resistant *Enterococcus* (VRE), or carbapenem-resistant *Enterobacteriaceae* (CRE). Thus, two patients with identical hospital exposures might have different outcomes: one’s intact microbiome fends off a potential invader, while another’s depleted flora allows it to proliferate. Studies have shown that patients on prolonged antibiotics accumulate higher loads of resistance genes in their gut microbiome, serving as a reservoir for potential transfer to pathogens [89].

Microbiome differences also affect how drugs are processed and the local gut environment during infection. For instance, certain gut bacteria can metabolize or inactivate antibiotics—meaning patient A’s microbiota might degrade a portion of an oral antibiotic dose, leading to lower systemic levels, whereas patient B’s does not. An example is the gut microbial enzyme β-glucuronidase, which can reactivate certain xenobiotic and endobiotics [90], or bacterial enzymes that modify drugs like digoxin [91]. Recent reviews highlight that gut microbes can modulate the pharmacokinetics and pharmacodynamics of antibiotics. For example, gut bacteria have been found to alter vancomycin’s absorption and metabolism, as well as influence the host’s immune response to the drug [92]. Additionally, vancomycin itself causes gut dysbiosis, eliminating many Gram-positive commensals and potentially opening the niche for VRE or *C. difficile*. The net effect is that two patients given the same antibiotic can experience different drug exposure and different collateral damage to their microbiome, translating into different outcomes and future risks.

There is also evidence that microbiome composition correlates with treatment outcomes in certain infections. For example, in hematopoietic stem cell transplant patients, a gut microbiome dominated by a single genus (low diversity) is associated with higher likelihood of bloodstream infections by MDR bacteria. In these patients, interventions to modulate the microbiome are being explored. One novel strategy is fecal microbiota transplantation (FMT) or targeted probiotics to decolonize resistant organisms. Early studies have shown FMT can successfully eradicate carriage of MDR Enterobacteriaceae or VRE in some individuals, thereby reducing subsequent infection risk. FMT was used in immunocompromised patients to clear gastrointestinal MDR colonization, resulting in significantly fewer MDR infections post-transplant [93]. Though still experimental, this highlights how manipulating a patient’s microbiome could improve outcomes by removing the “seed bed” for resistant infection. More broadly, the microbiome produces various metabolites that influence host immunity and pathogen growth. Short-chain fatty acids, for instance, can enhance immune barrier function and have been shown to reduce Salmonella invasion [94]. Differences in such microbiome-derived factors may alter how effectively a host contains an infection. Re-establishing a healthy microbiota is now seen as a potential therapy for fighting drug-resistant infections—for example, restoring gut flora diversity after antibiotics to prevent recurrent *C. difficile* or MDR organism colonization [95].

Patient-to-patient variability in microbiomes means that the same pathogen and antibiotic regimen might lead to different trajectories. One patient’s microbiome might suppress the pathogen or mitigate drug side effects, whereas another’s might be conducive to pathogen overgrowth or rapid resistance selection. This formed the basis for microbiome-aware antibiotic prescribing. Recent advances in metagenomics are enabling profiling of patients’ resistome [96] and tailoring antibiotics to minimize disruption. The goal is to choose regimens that treat the infection while sparing beneficial commensals, thereby reducing the emergence of resistance. Adjuvant therapies like probiotics [97], prebiotics [98], and synbiotics [99] are also being investigated to maintain or restore microbiome balance during antibiotic treatment. Though methodologies need standardization, these approaches hold promise for improving patient outcomes by leveraging the microbiome.

### 3.3. Pharmacokinetic Variability and Drug Exposure Differences

Not all patients process and distribute drugs in the same way. Pharmacokinetic (PK) variability—differences in how individuals absorb, metabolize, distribute, and excrete antibiotics—can lead to substantial differences in antibiotic concentrations achieved at infection sites. This variability is especially pronounced in critically ill patients and those with organ dysfunction [100]. Inadequate antibiotic exposure in a patient can foster treatment failure and even select for resistance. For instance, critical illness often causes an increased volume of distribution and enhanced clearance, which means a standard dose of antibiotic may get diluted or cleared faster, resulting in lower plasma and tissue levels than expected. If dosing is not adjusted, the patient might effectively be under-dosed despite receiving the “recommended” amount. A review of β-lactam PK in ICU patients noted that these pathophysiological changes frequently lead to subtherapeutic drug concentrations, treatment failure, and the development of antibiotic resistance when standard doses are used [101]. In other words, the infection sees just enough antibiotic to exert selective pressure but not enough to clear the bacteria, a recipe for resistance selection.

One classic example is the variability in vancomycin levels. Obese patients, or those with high clearance, may have very low trough levels if dosed routinely, which can result in persistent MRSA bacteremia that slowly acquires further resistance [102]. Similarly, in pediatric vs. adult patients, or men vs. women, or among different ethnic populations, genetic differences in liver enzymes can affect how quickly drugs like rifampicin or isoniazid are metabolized, altering effective exposure. If a fast-metabolizer patient gets rid of the drug too quickly, the bacteria have a better chance of surviving and potentially developing resistance mutations.

Pharmacokinetic variability is also evident in the outpatient community setting. A modeling study on *Streptococcus pneumoniae* and β-lactams examined the impact of “received dose” heterogeneity in the population [103]. Model simulation revealed that lower antibiotic doses consistently led to a higher prevalence of nonsusceptible strains over time, whereas higher doses suppressed the emergence of resistance to an extent. This underscores the principle that insufficient drug exposure is a driver of resistance. In the hospital context, this principle is seen when, for example, a patient with renal failure is under-dosed to avoid toxicity but then the drug levels are too low to clear the infection, enabling a resistant subpopulation to take over.

In addition to factors such as critical illness, patient adherence and pharmacological interactions substantially influence antimicrobial treatment outcomes and resistance development [104]. Suboptimal adherence—for example, discontinuing a prescribed 14-day antibiotic regimen prematurely at day 5—can lead to incomplete bacterial clearance and foster the emergence of resistant subpopulations. Studies have shown that patient knowledge and communication with healthcare providers significantly impact adherence, with misinformation and limited understanding of antibiotic use and AMR serving as major barriers [105]. Furthermore, pharmacokinetic variability due to drug–drug interactions can affect antibiotic exposure [106]. These findings underscore the importance of integrated communication strategies and individualized treatment planning to ensure appropriate antibiotic exposure and minimize resistance risk.

Patient-specific pharmacokinetics can cause large differences in the effective dose that bacteria experience. Inadequate exposure in one patient versus another can mean the difference between cure and the selection of a more resistant infection. Recognizing this, there is a push towards precision dosing—taking into account individual factors like organ function, weight, genetic metabolizer status, infection site to tailor antibiotic regimens. This personalized approach aims to ensure each patient gets sufficient drug exposure to eradicate the pathogen. It is hoped that by minimizing under-dosing, we not only improve patient outcomes but also reduce the emergence of resistance.

## 4. Connecting Microbial Heterogeneity and Host Variability: Implications for Treatment Outcomes

The intersection of bacterial phenotypic heterogeneity and host variability ultimately determines real-world treatment outcomes [107,108]. Consider a scenario: two patients are infected with the same strain of a multidrug-resistant *Pseudomonas*. Patient A has well-controlled diabetes and no immune deficits; Patient B is immunosuppressed post-transplant and has just completed a course of broad-spectrum antibiotics. In patient A, a high-dose antibiotic regimen (tailored to their normal renal function) combined with an intact immune system might succeed in clearing the infection—any persisters left after therapy can be mopped up by the immune response. In patient B, however, standard dosing might yield suboptimal drug levels (due to altered PK) and their impaired immune system cannot eliminate residual bacteria. Moreover, patient B’s recent antibiotic use may have wiped out competing gut flora, allowing any surviving *Pseudomonas* persisters to flourish. Thus, what was a cure in one patient becomes a persistent or relapsing infection in another. This example highlights that microbial and host heterogeneity are not independent factors but synergistic [109].

One concrete illustration is in cystic fibrosis lung infections. CF patients often develop *P. aeruginosa* infections that evolve into diverse biofilm communities with persister subpopulations. The CF lung environment is a unique host setting that further limits antibiotic penetration and blunts immune clearance [110]. The host microenvironment and bacterial lifestyle co-create tolerance so that standard lab susceptibility results have little correlation with clinical response. Treatment requires not just antibiotic selection based on MIC, but consideration of drug diffusion into mucus, activity in biofilm conditions, and possibly immunomodulators to assist clearance. Another example is prosthetic joint infection by *Staphylococcus*. Bacteria form biofilms on the implant (bacterial heterogeneity factor), and the patient’s immune response is relatively ineffective at that interface [111]. Even with prolonged antibiotics, a tiny reservoir of persisters on the implant can cause relapse months later—often the only cure is surgical removal of the device. In such cases, the clinical outcome depends on addressing both the microbe’s phenotypic defenses and the host context.

Treatment failures often involve susceptible pathogens when considered in vitro, and the reasons lie in phenotypic resilience of the bacteria plus host/environment factors that impede killing [112]. It stressed that focusing only on pathogen genotype misses the “forest for the trees”—factors like persister formation, biofilm growth, and suboptimal host immune function are major contributors to failure. Likewise, clinicians are recognizing that a shift toward a patient-centered approach is needed in AMR management [113]. Instead of only asking “which pathogen and what drug is it resistant to?”, we must also ask “what patient factors might influence the outcome here?” For instance, a Difficult-to-Treat Resistance (DTR) pathogen in a robust host with prompt source control might be overcome, whereas even a “moderately” resistant bug in a frail host could be lethal.

The interplay between microbial heterogeneity and host variability also has evolutionary implications. A patient with suboptimal antibiotic doses or weak immunity effectively becomes a training ground for the pathogen to develop greater resistance. In such an environment, even phenotypic tolerance mechanisms can facilitate permanent resistance: for instance, a persister state allows some bacteria to survive long enough to acquire a true resistance mutation during prolonged exposure [114]. This has been observed in experiments where tolerance precedes genetic resistance under intermittent antibiotic exposure. Similarly, a host unable to clear an infection will be subjecting the bacteria to extended antibiotic courses, increasing selective pressure for resistance. On a population level, this is why antimicrobial resistance is often prevalent in settings with many immunocompromised patients. Those patients are not only more likely to need broad-spectrum drugs, but once a resistant strain emerges, the host is less likely to eradicate it, allowing it to spread.

## 5. Emerging Insights and Future Directions

Researchers are developing laboratory tests that go beyond the standard MIC to detect persisters and heteroresistance in clinical isolates. For example, tolerance/persistence assays measure the survival of bacteria after exposure to a high antibiotic dose to quantify the persister fraction [32]. Heteroresistance can be unmasked by population analysis profiling—exposing cultures to antibiotic gradients and looking for subpopulations that grow. While these tests are not yet routine, they are crucial for identifying patients at risk of treatment failure despite susceptible lab results. Rapid single-cell imaging methods and microfluidic devices are also in development to observe bacterial responses to antibiotics in real time, which could flag phenotypic tolerance within hours [115]. The challenge is to make such diagnostics fast and user-friendly for clinical labs. A rapid, single-cell diagnostic platform using droplet microfluidics was developed for both pathogen identification (ID) and antimicrobial susceptibility testing (AST), achieving results directly from urine samples in as little as 30 min. By detecting 16S rRNA with peptide nucleic acid (PNA) probes and monitoring single-cell growth under antibiotic exposure, the system enables high-throughput, sensitive, and scalable diagnostics [116]. This technology holds significant clinical promise by drastically reducing the time to targeted therapy, minimizing reliance on empirical broad-spectrum antibiotics, and helping curb the spread of multidrug-resistant pathogens.

There is a growing focus on finding compounds that can either kill persister cells or prevent their formation. Some strategies aim to “wake up” persisters (push them out of dormancy) so that conventional antibiotics can then kill them [117]. Examples being explored include metabolic stimuli and alarmone signaling inhibitors. A study found that stimulating the tricarboxylic acid (TCA) cycle in P. aeruginosa with fumarate made dormant cells more susceptible to tobramycin, even in CF patient sputum models [118]. Another approach is using phage therapy: certain bacteriophages have enzymes that degrade biofilm matrix or can infect stationary-phase cells, potentially helping to clear persisters [119]. Although phage therapy is not mainstream in most countries, case reports of phage–antibiotic combinations show promise in clearing refractory MDR infections [120]. Adjunctive enzymes or chelating agents that break biofilms are also under investigation to improve antibiotic penetration [121,122]. From the host perspective, increasing the immune response to persisters is another angle—for instance, therapies to enhance macrophage autophagy might help clear intracellular persister reservoirs in diseases like TB [123].

Given that patient immune status is so pivotal, therapies that bolster the host’s infection-fighting ability are being pursued. Granulocyte colony stimulating factor (G-CSF) is sometimes used in neutropenic patients to increase white cell counts during infection [124]. Novel immunotherapies, such as monoclonal antibodies against bacterial toxins or virulence factors, can neutralize pathogens and give the immune system a better chance [125]. Checkpoint inhibitors used in cancer immunotherapy have even been speculated to improve immune response to infection [126]. Additionally, reconfiguring the microbiome is a future direction—for example, prophylactic probiotics in high-risk patients to reduce colonization by MDR organisms or post-antibiotic microbiome restoration [127]. FMT is being tested to decolonize patients carrying CRE or VRE in their gut [128]. If successful, such approaches could become standard in preventing recurrent infections by those organisms.

On the pharmacokinetic front, there is a push for precision medicine in antibiotic dosing. This includes routine use of antibiotic level monitoring in ICU patients, software that uses patient-specific data to predict the right dose [129], and possibly point-of-care tests of drug levels. It is increasingly recognized that research into personalized antibiotic therapy is needed to improve outcomes and reduce resistance selection. For example, if a rapid test could tell us a patient’s β-lactam level and the pathogen’s MIC, dosing could be adjusted in real time to ensure effective concentrations. Adaptive trial designs are also being considered, where antibiotic therapy is modified based on early patient responses or pathogen load kinetics [130]. All these aim to avoid the perils of under-dosing (leading to resistance) and over-dosing (causing toxicity or collateral damage).

A truly integrated approach is on the horizon, where diagnostic and therapeutic decisions consider both microbial genotypic/phenotypic data and host biomarkers. For instance, measuring host inflammatory markers could help determine if an infection is truly cleared or if persisters likely remain. Machine learning models are being developed that take into account patient features alongside pathogen features to predict outcomes and recommend therapies. The resulting concept of a “host–pathogen index” could guide clinicians [131].

AI is emerging as a powerful tool to tackle the complexity of antimicrobial resistance at both the microbial and patient levels. Machine learning and deep learning algorithms can integrate diverse data streams—genomic profiles, transcriptomic patterns, electronic health records, microbiome data, and pharmacokinetic parameters—to improve prediction of resistance, optimize antibiotic selection, and even forecast treatment outcomes in individual patients [132,133,134]. In the context of cell-to-cell variability, AI models can help disentangle subtle genomic and transcriptomic signatures that drive phenotypic heterogeneity, including persistence and heteroresistance, which are often invisible to conventional diagnostics. Clinically, AI-driven decision-support systems hold promise for personalizing antimicrobial therapy, ensuring that drug choice, dose, and duration are tailored not only to the pathogen but also to patient-specific factors such as immune status and comorbidities. Looking forward, integrating AI into AMR management could transform antibiotic resistance into a precision medicine discipline, bridging the gap between laboratory complexity and bedside decision-making [135].

In conclusion, contemporary research makes clear that antimicrobial resistance is a multifactorial challenge. Cell-to-cell variability allows bacterial populations to hedge against antibiotics, while patient-to-patient variability means the same infection can play out in vastly different ways. Effective solutions will require a holistic approach: one that pairs novel anti-persister or anti-biofilm drugs and smart antibiotic use with strategies to strengthen host defenses and personalize therapy. By appreciating the roles of phenotypic heterogeneity and host differences, clinicians and researchers can design better interventions—from more predictive diagnostic tests to tailored treatment regimens—ultimately improving outcomes in the face of resistant infections. The past decade has seen major strides in this understanding, and the next decade will likely bring these insights into clinical practice, bridging the gap between bench and bedside in the fight against AMR.

## Data Availability

No new data were created or analyzed in this study. Data sharing is not applicable to this article.
